# Culturally specific versus standard group cognitive behavioral therapy for smoking cessation among African Americans: an RCT protocol

**DOI:** 10.1186/2050-7283-1-15

**Published:** 2013-08-21

**Authors:** Monica Webb Hooper, Ramona Larry, Kolawole Okuyemi, Ken Resnicow, Noella A Dietz, Robert G Robinson, Michael H Antoni

**Affiliations:** Sylvester Comprehensive Cancer Center, University of Miami, PO Box 248185, Coral Gables, FL US; Miller School of Medicine, Sylvester Comprehensive Cancer Center, University of Miami, 1120NW 14th Street, Miami, FL US; University of Minnesota, 717 Delaware Street SE, Minneapolis, MN US; University of Michigan, 1415 Washington Heights, Ann Arbor, MI US; 3495 Hidden Acres Drive, Doraville, GA US

**Keywords:** Smoking cessation, African Americans, Culturally specific, Cognitive behavioral therapy

## Abstract

**Background:**

African American smokers experience disproportionately higher rates of tobacco-related illnesses compared to Caucasians. It has been suggested that interventions targeted to specific racial/ethnic groups (i.e., culturally specific) are needed; however, the literature examining the efficacy of culturally specific interventions is equivocal. Moreover, there are few descriptions of methods used to create these interventions. The main aim of this study is to test the efficacy of a culturally specific smoking cessation intervention among African Americans.

**Methods/Design:**

A 2-arm randomized controlled trial (RCT) will be conducted to assess the efficacy of a culturally specific group cognitive behavioral therapy (CBT), compared to standard group CBT among treatment-seeking smokers from the community. Participants in both conditions receive the transdermal nicotine patch (TNP) for 8-weeks. We intend to randomize at least 247 adult smokers who self-identify as African American into the trial. Enrolled participants are block randomized into one of two groups: Standard group CBT (control) or a culturally specific group CBT (CS-CBT). Groups are matched for time and attention, and consist of eight sessions. The primary outcome variable is 7-day point prevalence abstinence (7-day ppa). Smoking status is assessed at the end-of-counseling (EOC), and 3, 6, and 12-month follow-ups, with self-reported abstinence verified by saliva cotinine. We hypothesize that the CS-CBT condition will produce significantly greater smoking cessation rates compared to the control condition. We also expect that this effect will be moderated by acculturation and ethnic identity, such that the CS-CBT will show the greatest effect on cessation among participants who are less acculturated and have greater ethnic identity.

**Discussion:**

Answering the fundamental question of whether culturally specific interventions lead to incremental efficacy over established, evidence-based approaches is of utmost importance. This study will have implications for the development and implementation of smoking cessation interventions among African Americans and other racial/ethnic minority groups.

**Trial registration:**

NCT01811758

## Background

### Significance

In 2011, 19.4% of African American adults were current smokers (CDC 2012). Although comparable to the overall population, the prevalence among low-income African Americans is notably higher (40%-60%) (Delva et al. 2005). In addition, African American smokers experience disproportionate rates of smoking-related disease and death compared to other racial-ethnic groups (Park et al. 2011;American Cancer Society 2007). Smoking characteristics differ between African Americans and Caucasians, some of which may help explain these disparities in health. It is known that compared to Caucasian smokers, African Americans are more likely to smoke mentholated brands, have higher serum cotinine concentrations per cigarette smoked (Caraballo et al. 2011), are less likely to use evidence-based cessation treatments and are less likely to achieve cessation (Fu et al. 2008;Trinidad et al. 2011). The latter points may in part be attributable to the lower likelihood of receiving appropriate smoking cessation advice from providers (Lopez-Quintero et al. 2006) and the failure of prior interventions to address ethno-cultural factors that may limit their effectiveness. Needed are evidence-based interventions that specifically target African American smokers. The purpose of this study is to evaluate the incremental efficacy of addressing unique ethno-cultural factors within the context of an established cognitive behavioral therapy for smoking cessation in a sample of African Americans.

### Cognitive behavioral therapy for smoking cessation

Cognitive behavioral therapy (CBT) for smoking cessation and relapse prevention have established efficacy (Fiore et al. 2008;Song et al. 2010). CBT for smokers includes a focus on coping skills training, and has efficacy at least comparable to pharmacotherapy (Fiore et al. 2008), with greater cost-effectiveness (Cromwell et al. 1997). Group-based CBT is particularly efficacious, and provides social support, positive reinforcement, psychoeducation, and cognitive behavioral strategies for coping and stress management (Stead & Lancaster 2005). Little previous research has examined group smoking interventions with CBT components among African American. Two studies found evidence for efficacy when compared to assessment only and minimal self-help (Murray et al. 2001;Knight 2004). Only one trial compared CBT to a time-and-attention matched control condition (Webb et al. 2010), which was the first study to demonstrate that CBT was causally related to smoking cessation among African Americans. However, because the intervention was delivered using a standard (i.e., non-culturally specific) format, it did not address the unique ethno-cultural characteristics of African American smokers. Indeed, this standard intervention had lower efficacy among the subgroup of smokers with traditional African American values and cultural practices (Webb Hooper et al. 2012).

### Culturally specific smoking cessation interventions

Culturally specific approaches to behavior change integrate race, ethnicity, social factors, culturally traditional norms and values, and behavior patterns into the core of interventions. Such interventions have been referred to using various terms, including culturally sensitive, targeted, tailored, and competent. This study uses the term culturally specific to convey that the intervention is designed for a specific ethno-cultural group (i.e., African American smokers), yet may not apply equally to all members. Models of culturally specific interventions targeting African Americans emphasize the significance of framing the content and presentation within a context that is appropriate for the group (Kreuter et al. 2002;Resnicow et al. 1999). Resnicow and colleagues (1999) described two components of culturally sensitive interventions, surface and deep structure. The goal of surface structure is to adapt the presentation of interventions to facilitate acceptability, receptivity, and capture attention (e.g., race-matched images). In contrast, deep structure adapts the intervention content by addressing meaningful historical, socio-cultural, environmental, and psychological factors (e.g., collectivism, religion, and racism). The intervention in the current study includes both surface and deep structure elements.

More research is needed to test culturally specific interventions for African American smokers. A few studies have compared culturally specific self-help materials to standard control groups, and found a preference for the culturally specific booklets (Webb 2009;Orleans et al. 1998) and greater quit attempts among participants in the culturally specific condition (Orleans et al. 1998;Nollen et al. 2007). These studies did not find smoking cessation differences between conditions, which is the primary goal of most interventions. It is possible that adapting existing interventions with demonstrated efficacy among African American smokers will add incremental efficacy to outcomes. This assertion is supported by previous research in the psychotherapy literature indicating that culturally specific interventions are more effective than traditional interventions, and that this effect is positively associated with the extent of specificity (Smith et al. 2011).

### Consideration of ethno-cultural individual differences

Because race is not equivalent to a monolithic culture, culturally specific smoking cessation interventions may benefit some smokers, but not others (Webb 2008). It is important to consider individual differences in acculturation (i.e., the extent of engagement in one’s traditional cultural beliefs, values, and practices versus adoption of the dominant culture) and ethnic identity (i.e., identification and affiliation with one’s ethnic group), as these factors may affect outcomes. African American smokers are likely to be less acculturated compared to African American non-smokers (Klonoff & Landrine 1999;Landrine & Klonoff 1994). Acculturation is also predictive of culturally specific intervention receptivity. Webb (2008) found that less acculturated African American smokers preferred culturally specific written materials, while those higher on acculturation preferred standard materials. Ethnic identity also has the potential to influence outcomes following culturally specific interventions. Resnicow et al. (2009) found that tailoring a self-help nutrition newsletter on ethnic identity resulted in improved fruit and vegetable intake among Afrocentric African Americans. No previous research has examined the role of ethnic identity in culturally specific interventions among smokers. This study, however, will explore the possibility that culturally specific CBT will be more efficacious among smokers with greater ethnic identity.

### The present study

This study will address an important gap in the literature by answering a fundamental question regarding the use of culturally specific interventions among African American smokers in a randomized controlled trial. Previous research suggests a positive role of cultural specificity for process outcomes in self-help trials, but no studies have demonstrated a significant effect on smoking cessation within more potent interventions (e.g., group CBT). We hypothesize a main effect of cultural specificity, such that CS-CBT will result in greater smoking cessation rates compared to standard CBT. We also expect to find a main effect of time, such that the CS-CBT condition will result in greater cessation rates through 12-months. We do not anticipate an intervention × time interaction. Our exploratory analyses will consider the moderating roles of acculturation and ethnic identity on smoking cessation outcomes. Specifically, we expect that less acculturated participants (i.e., highly engaged in traditional African American culture) and those with greater ethnic identity will show the greatest cessation rates if they are in the CS-CBT condition.

## Design and method

**Figure 1:**
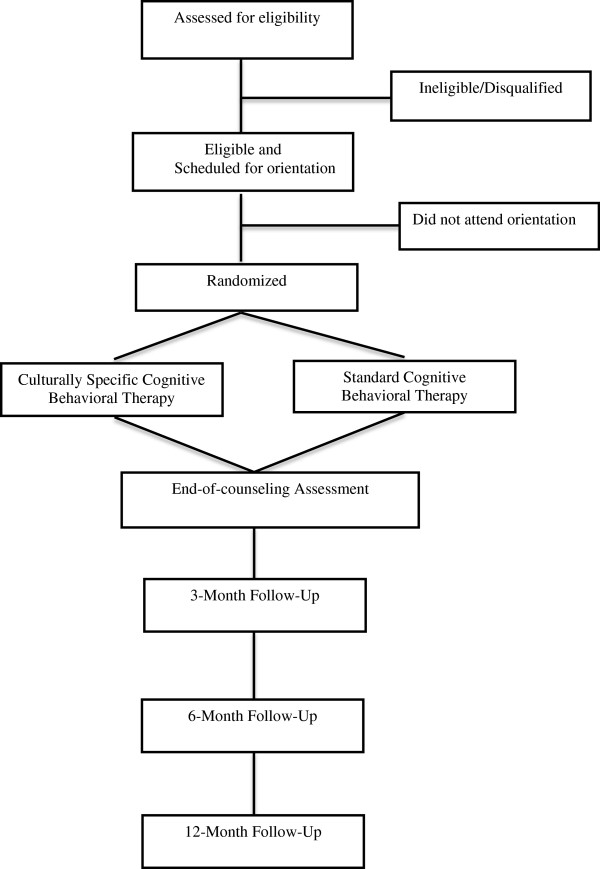
**RCT flowchart.** Illustration of study design and participant flow.

This phase 1 efficacy study is a 2 (intervention) × 4 (time) mixed factorial design with cotinine-confirmed cessation as the primary outcome. Factor 1 is the type of intervention: culturally specific CBT (CS-CBT) versus standard CBT (control), both supplemented by 8-weeks of transdermal nicotine patch (TNP) therapy. CBT in both conditions consists of cognitive and behavioral strategies guided by evidence-based smoking cessation and relapse prevention models (Marlatt & Gordon 1985). The key difference between conditions is whether the intervention is culturally specific. The culturally specific components (e.g., discussion of race and smoking, race-matched clinicians, and an emphasis on religion/spirituality) are those described in the literature and our prior research. Factor 2 is time: End-of-counseling (EOC), and 3, 6, and 12-month post counseling assessments. This study includes a controlled, internally valid, experimental test of the efficacy of CS-CBT among African American smokers. Figure 1 illustrates the flow of participants through the trial.

### Participants and recruitment

Participants will be 247 African American tobacco smokers recruited from the community. We developed a comprehensive recruitment plan, consisting of advertisements on public transportation, partnering with healthcare organizations with large racial minority clientele, and street outreach (directly talking with people in predominantly Black neighborhoods and visiting local businesses). Participants are considered eligible if they: (1) self-identify as African American; (2) currently smoke ≥ 5 cigarettes/day or have an expired CO level of ≥ 8 ppm; (3) are ages 18–65; (4) are able to read 5^th^-6^th^ grade English; (5) have permanent contact information; (6) are able to attend clinic sessions (transportation costs are reimbursed); and (7) are motivated to quit smoking (rated as a 6 on a 1–10 scale). We exclude those who are currently receiving any type of cessation, alcohol or illicit drug treatment, pregnant/breastfeeding, or diagnosed with an acute cardiac or respiratory condition. Ineligible participants are referred to the Florida QuitLine.

### Randomization

Eligible participants are randomly allocated using a 1:1 ratio in blocks of 50 to one of the two conditions, CS-CBT or control. The unit of randomization is the individual. Eligible participants receive a tentative random assignment and are scheduled for an orientation session, with only those who attend and provide written informed consent enrolled in the study. We schedule up to 25 participants per group to ensure 8–12 tentatively assigned participants are enrolled/consented into the study.

### Procedures

Prior to orientation, welcome letters containing the schedule of group sessions and directions are mailed. Reminder calls are placed to facilitate continued interest in the study. Participants attend orientation, eight clinic sessions, and 3, 6, and 12-month follow-up assessments.

### Interventions

#### Orientation and intervention sessions

Participants attend a 60-minute orientation before the start of the clinic sessions. They learn the background, purpose, format, and procedures of the study; provide informed consent; complete baseline measures; learn about TNP therapy and receive their first patch (the remainder of the patches are provided throughout active treatment); and provide breath carbon monoxide (CO) and saliva samples for cotinine analysis. We also cover smoking and health, self-motivation, and goal setting. In the CS-CBT condition, we explain that the group is framed within a cultural context, designed to emphasize African American race/ethnicity and cultural issues.

In accordance with previous research (Webb et al. 2010;Brandon et al. 1995) participants in both conditions are asked to reduce their smoking by one-half on the day before the first clinic session and to abstain completely from smoking on the first day of actual group treatment (session 1). They are also instructed to begin patch use on the morning of the first day of treatment (session 1; the target quit day) with the patch provided at orientation. This aspect of the protocol is unique from other cessation approaches that set the quit day several weeks post beginning the intervention. However, this evidence-based protocol has been successful in previous studies [e.g., Webb et al. 2010; Brandon et al. 1995 and is also the format we use in our ongoing cessation clinic. Participants who do not quit on the target day are encouraged to make a quit attempt by the third treatment session.

Participants in both conditions meet eight times over four consecutive weeks. Four sessions occur during week 1, two during week 2, and one weekly booster session during weeks 3 and 4. Depending on group size, the duration of sessions is 90–120 minutes in both conditions. We anticipate 8–16 participants per group. Co-therapy pairs of masters or bachelor’s level interventionists are trained to conduct sessions for one condition (CS-CBT or control) and supervised by the principal investigator (PI). Groups are held in a laboratory-based clinic. Incentives include $40 at session 1, $20 at session 5, $50 at session 8, $50 at the 3-month assessment, $70 at the 6-month assessment, and $70 at the 12-month assessment. Participants also receive $5 per session for transportation/parking and light refreshments at each session.

#### Intervention conditions

1. *Group cognitive behavioral therapy for smoking cessation (control):* The intervention in this condition is based on standard cognitive and behavioral strategies, supplemented by TNP therapy. A previous study testing this intervention in an African American sample found 7-day ppa rates of 70% at the EOC, 52% at the 3-month follow-up, and 46% at the 6-month follow-up post counseling (Webb et al. 2010). Session content is displayed in Table 1, and includes the benefits of quitting, nature of nicotine addiction, nicotine withdrawal, identification of “high risk” situations, motivation, coping skills, stress and negative affect, decision making, alcohol use, weight control, social support, behavioral contracting, and relapse-prevention. To enhance the external validity of the intervention, the co-therapy team is not race-matched (at least one interventionist is non-African American/Black).
2. *Culturally specific group cognitive behavioral therapy for smoking cessation (CS-CBT):* The intervention is the standard CBT program with an emphasis on African American culture (Table 1). Each session focuses on specific aspects of traditional African American culture. The CS topics were selected based on our previous qualitative research (Webb et al. 2007), and established models e.g. (Robinson et al. 1992). Topics include deep structure: Distrust for biomedical research; race and smoking, race-based statistics related to nicotine replacement/medication concerns; religion/spirituality; family/collectivism; unique stressors; racism/discrimination, depression among African Americans; co-morbid addiction; neighborhood/environmental influences; targeted tobacco marketing; menthol cigarettes; race-specific weight issues and concerns; and working as a community against the tobacco industry. Surface structure is also included: Interventionists are race-matched, second-person phrases (e.g., us, we) are used throughout, and the daily agenda includes African American quotations or proverbs.

**Table 1:** **Overview of the interventions**

	CBT	CS-CBT
**Orientation**	Study explanation, structure of sessions and TNP, informed consent, health and smoking, research participation, goal setting, baseline assessment, breath carbon monoxide (CO) and saliva samples.	Same as in the CBT condition. Race and smoking, views on research participation, distrust for biomedical research, concerns about nicotine replacement, goal setting, baseline assessment, breath CO and saliva samples, race-matched clinicians (RMC).
**Session 1**	Review quit plan, positive reinforcement (PR), reasons for quitting, nicotine addiction, introduction to coping response training model, smoking and motivation, TNP use, plan for next 24 hours, behavioral contracting (BC), CO.	Standard CBT. Meaning of being African American, tobacco and African Americans, menthol, RMC.
**Session 2**	Review quit plan progress, PR, benefits of quitting, coping skills, plan for next 24 hours, BC, CO.	Standard CBT. Spirituality and religion in the African American community, RMC.
**Session 3**	Review quit plan progress, PR, stress management, alcohol and smoking, high cost of smoking, plan for next 48 hours, perceived benefits of quitting, BC, CO.	Standard CBT. Stressors unique to African Americans, discrimination and racism, deep breathing exercises, co-morbid addiction, little cigars and blunt use, RMC.
**Session 4**	Review quit plan progress, PR, negative affect and smoking, cognitive restructuring, decision making, plan for next 72 hours, BC, CO.	Standard CBT. Traditional African American values, buddy system, deep breathing exercise, RMC.
**Session 5**	Review quit plan progress, PR, review personal high-risk situations, relapse prevention, responding to lapses, plan for next 72 hours, BC, CO.	Standard CBT. Mood and depression among African Americans, deep breathing exercise, RMC.
**Session 6**	Review quit plan progress, PR, individual high-risk situations, noted benefits of quitting, weight and smoking cessation, minimizing weight gain, relapse prevention, plan for next 7 days, BC, CO.	Standard CBT. Weight and African Americans, smoking and weight concerns (super-gainers), minimizing weight gain (physical activity with limited resources, healthy food choices within soul food diet, recipes), deep breathing, RMC.
**Session 7**	Review quit plan progress, PR, planning for group termination, a new lifestyle, social support, plan for next 7 days, BC, CO.	Standard CBT. Life in your neighborhood, environmental influences, gaining freedom from smoking, deep breathing exercise, RMC.
**Session 8**	Review quit plan progress, PR, reflect on group experience, long-term trip-ups, review of coping response training, withdrawal, relapse prevention, TNP schedule, follow-up procedures, BC, CO and saliva samples.	Standard CBT. Body as a temple, resources, mobilizing the African American community against the tobacco industry, deep breathing exercise, RMC.

Note: Topics that are not explicitly culturally specific are discussed within this context.

#### Transdermal nicotine patch therapy (TNP)

TNP has demonstrated efficacy in multiple trials (Silagy et al. 2000), is available over-the-counter, and is safe and effective for smoking cessation (Shiffman et al. 2002) without monitoring by a physician. Consistent with (Webb et al. 2010) and (Fiore et al. 2008), participants are prescribed four weeks at 21 mg, two weeks at 14 mg, and two weeks at 7 mg (Doses are adjusted according to smoking history).

#### Training and intervention fidelity

The interventionists are trained by the PI. Training includes relevant readings (smoking, cessation and relapse prevention, cognitive behavioral therapy, motivational interviewing), observation of two therapy groups, minimal contributions in the role of therapist, and finally, fully conducting sessions with weekly PI supervision. Detailed intervention manuals are followed closely and participants receive a daily agenda. Interventionists do not cross over to prevent contamination. Most sessions are audio recorded and will be coded using a 10-item scale for adherence [see Webb et al. 2010 by two independent evaluators. Codes will be transformed into percentages indicating the degree of protocol adherence.

### Measures

#### Baseline

Measures include demographics, smoking history, and nicotine dependence (Heatherton et al. 1991) (Table 2). We also assess perceived stress (Cohen et al. 1983), depressive symptoms (Radloff 1997), and decision making. Particularly relevant for African American smokers, we include assessments of acculturation (Klonoff & Landrine 1999), ethnic identity (Davis et al. 2010), expectancies for culturally specific interventions, and perceived ethnic discrimination (Landrine et al. 2006). We also record height and weight. Participants also complete an “In Case I Move Form” as a method of tracking participants via relatives or friends.

**Table 2:** **Constructs and measures**

Assessment points						
Measure	Baseline	Intra-treatment	End-of-counseling	3-month follow-up	6-month follow-up	12-month follow-up
Attendance	✓	✓	✓	✓	✓	✓
Demographics	✓					
Smoking history	✓					
Nicotine dependence	✓					
Height	✓					
Weight	✓	✓	✓	✓	✓	✓
Perceived stress	✓		✓			
Depressive symptoms	✓		✓			
Acculturation	✓					
Ethnic Identity	✓					
Expectancies for culturally specific Interventions	✓					
Decision making	✓					
Perceived ethnic discrimination	✓					
Nicotine withdrawal		✓	✓			
Smoking urges		✓	✓			
TNP use		✓	✓			
Intervention ratings			✓			
Time-line follow-back (smoking pattern)			✓	✓	✓	✓
Therapist satisfaction			✓			
Carbon monoxide	✓	✓	✓	✓	✓	✓
Cotinine*	✓			✓	✓	✓

Note: *At baseline, saliva for cotinine is collected from all participants. Cotinine samples are not collected at the EOC because participants are still using nicotine replacement. At follow-ups, saliva for cotinine is collected from self-reported abstainers only.

#### Intra-treatment

Attendance, TNP utilization, and weight are recorded at each session. Participants also self-report, via monthly telephone follow-ups, utilization of 14mg, and 7mg patches across the full 8-weeks. The Minnesota Withdrawal Scale (Hughes & Hatsukami 1986) is administered as an indicator of nicotine withdrawal, and the Questionnaire of Smoking Urges-Brief (Sanderson Cox et al. 2001) assesses urges to smoke.

#### End-of-counseling

Participants evaluate the intervention, using the Intervention Rating Questionnaire (Webb et al. 2010), and complete measures of perceived stress, depressive symptoms, nicotine withdrawal, and therapist satisfaction (Oei & Green 2008) (Table 2). Self-reported smoking status since the target quit date is assessed using the time-line follow-back (TLFB) procedure, which reconstructs the participant’s smoking pattern since the target quit date (Brown et al. 1998;Sobell & Sobell 1992).

#### Follow-up (3, 6, and 12-months)

Measures completed at each “reunion meeting” include the TLFB (Brown et al. 1998;Sobell & Sobell 1992), smoking status and use of other tobacco products and pharmacotherapy, and weight (Table 2).

#### Bio-verification

Smoking status is confirmed biochemically. Cotinine assays are collected at the orientation meeting (for a baseline level before cessation) and at the in-person 3, 6, and 12-month follow-ups (for self-reported quitters). Cotinine samples, using a cut point of 7 ng/ml, will determine smoking status (Abrams et al. 1987;Etter et al. 2000). The CBT protocol requires that breath carbon monoxide (CO) samples be collected at each session to provide participants with immediate feedback. Breath CO readings of at least 8 ppm will distinguish smokers from nonsmokers at the EOC, and have been found to be a sensitive method of determining smoking status (Benowitz et al. 2002). As recommended by (Benowitz et al. 2002), follow-up abstinence rates will be calculated separately for both self-report and biochemical findings.

#### Outcome variables

The primary outcome variable is 7-day ppa, assessed at the EOC, 3, 6, and 12-month follow-ups. Seven-day ppa refers to no smoking (not even a puff) for the past seven days (Hughes et al. 2003). Secondary outcomes include 24-hour (no smoking in the past day) and 28-day continuous abstinence (no smoking over the past 4 weeks).

### Data analyses

#### Sample size and power

Sample size was determined based on Webb et al. (2010) and the formula provided in (Diggle et al. 2002) (p. 31). Webb et al. 2010 found that 51% of participants who received standard CBT reported 7-day ppa at the EOC, and about a 20% relapse rate at 3-months and 6-months. In the CS-CBT pilot study, we found 75% 7-day ppa at the EOC. Assuming a 23% reduction at 3-months, four assessments, with a within-subjects correlation of .60, 65 participants per group will yield power = .80% with a two-sided significance level of 5%. We conservatively anticipated 30% 7-day ppa at 12-months in the CS-CBT condition, and 14% in the control condition. To examine acculturation and ethnic identity as moderators controlling for covariates (e.g., group, sociodemographic factors, etc.), the planned regression analyses require a sample of 124. Thus, the final N is 150 (completing all assessments).

#### Statistical analyses

Preliminary analyses will include graphics/plots, and descriptive statistics. We will compute frequencies and proportions for retention and baseline characteristics, and use t-tests and chi-squared tests to evaluate differences. Alpha will be set to .05, and adjusted for multiple comparisons. Missing values will be handled with appropriate methods (Little & Rubin 2002). Outcome analyses will be conducted with (a) an intent-to-treat (ITT) approach, in which participants with missing data are assigned the status of smoker, and (b) a “per protocol” approach, which will include participants who complete all aspects of the study; (orientation, ≥ four intervention sessions, and all follow-ups). Within-time logistic regressions will determine the odds of abstinence at each assessment, comparing CS-CBT to control. Generalized linear mixed modeling (GLMMs) will examine between-group cessation rates over time, including main effects and interactions, and accounting for nesting within groups. A pattern-mixture analysis will examine whether intervention effects differ according to patterns of missing data (e.g., those who complete only one follow-up). Hierarchical logistic regression will be conducted to explore ethno-cultural predictors of cessation.

### Ethics and safety

This study is being conducted with University of Miami Institutional Review Board approval. We undergo careful screening to attempt to identify respondents who are not appropriate for the study due to medical concerns that preclude TNP use (e.g., pregnant women, acute cardiac events). During the 1-year duration of the trial, participants may contact the research team in the event of an adverse event. During orientation, participants are advised to seek prompt medical attention in the case of severe side effects from the TNP or other unexpected emergency. Over the course of the intervention and follow-ups, nicotine withdrawal is monitored, in addition to the discussion of medical symptoms.

## Discussion

This RCT is the first to test the efficacy of a group-based, culturally specific CBT among African American smokers. It is also the first study to explore ethno-cultural factors as predictors of the intervention effect. Previous research has attempted to develop and test culturally specific smoking cessation interventions. In this regard, the notion of cultural specificity is not inherently innovative. However, our approach is innovative, as no previous study has adapted CBT to target African American smokers. Second, the CS-CBT is based on theoretical models and existing evidence. Third, we consider the role of individual-difference cultural factors as predictors. And, finally, the rigorous design will allow us to isolate the effect of cultural specificity per se by controlling for possible confounding factors (i.e., treatment intensity and duration). Moreover, the methodological limitations of the extant literature preclude an answer to the fundamental question of whether a focus on ethno-cultural factors has incremental benefits for smokers.

We acknowledge the limitations of this study. The sample consists of treatment-seeking, highly motivated smokers who likely differ from smokers less interested in cessation or those who would not be attracted to group interventions. The sample is also drawn from South Florida, and may not represent smokers in other geographic locations. Thus, we will not be able to generalize to other sub-groups of African American smokers. Study retention is a potential concern, which we attempt to address through incentives and relatively aggressive follow-up strategies (including phone calls, collateral contacts, text messages, mailed letters, and home visits). However, we successfully retained about 70% of African American smokers in a similar group intervention trial using less intensive methods (Webb et al. 2010).

Overall, this trial will answer important, unanswered questions that have the potential to transcend the smoking cessation literature into other areas of health behavior change. If our hypotheses are supported, our culturally specific approach may be used to modify and enhance established traditional intervention approaches, with the ultimate goals of cancer prevention and reducing smoking-related health disparities.
